# Development and Validation of an LC-MS/MS Method for the Determination of *Alternaria* Mycotoxins in Hepatic Tissue

**DOI:** 10.3390/toxins18020077

**Published:** 2026-02-02

**Authors:** María García-Nicolás, Alicia Navarro-Botia, Natalia Arroyo-Manzanares, Pilar Viñas

**Affiliations:** Department of Analytical Chemistry, Faculty of Chemistry, Regional Campus of International Excellence “Campus Mare Nostrum”, University of Murcia, E-30100 Murcia, Spain; maria.garcia66@um.es (M.G.-N.); pilarvi@um.es (P.V.)

**Keywords:** mycotoxins, *Alternaria* toxins, biomonitoring, liver, human, animals

## Abstract

The presence of *Alternaria* mycotoxins in hepatic tissue of both human and animal origin remains unexplored. This work describes the development of an analytical method based on salt-assisted liquid–liquid extraction (SALLE) and ultrahigh-performance liquid chromatography coupled to quadrupole time of flight mass spectrometry (UHPLC-QTOF-MS) for the determination of six main *Alternaria* mycotoxins and related metabolites. Sample treatment was fully optimized, including sample mass, extraction solvent, and volume and sodium chloride mass. The method was validated, achieving calibration curve R^2^ values above 0.99 and limits of detection between 0.01 and 1.46 µg kg^−1^. Moreover, satisfactory trueness (apparent recoveries between 84% to 111%) and precision (RSD values below 10%) were achieved, complying with EU requirements. Matrix effects in terms of signal suppression/enhancement varied between 53% for TeA and 78% for AME. Applied to real liver samples (20 human and 20 animal), alternariol monomethyl ether (AME) was found in pig liver, while alternariol (AOH) and tentoxin (TEN) were found in human forensic liver tissues. No other *Alternaria* mycotoxin metabolites were detected. This methodology is the first validated approach for determining *Alternaria* mycotoxins in liver tissue.

## 1. Introduction

Mycotoxins are a diverse group of toxic secondary metabolites produced by filamentous fungi belonging to genera such as *Penicillium*, *Aspergillus*, *Fusarium*, and *Alternaria* [1]. A wide range of agricultural commodities, including cereals, fruits, vegetables, and their processed derivatives, are commonly contaminated with these toxic compounds [2]. As a result, both humans and animals frequently consume them as part of their diets. Numerous environmental factors influence the biosynthesis, toxicity, and prevalence of mycotoxins, including, but not limited to, temperature, humidity, water activity, and nutrient availability [3]. However, the complex interactions among these factors and mycotoxin production remain poorly understood, posing a significant challenge for risk assessment and mitigation.

Mycotoxins produced by *Alternaria* species have attracted increasing scientific attention in recent years as they are prevalent in a wide range of food products, both fresh and processed [4]. Among the numerous *Alternaria* toxins, six have been identified as being of toxicological importance and comprise the focus of this study: alternariol (AOH), alternariol monomethyl ether (AME), tenuazonic acid (TeA), tentoxin (TEN), altenuene (ALT), and altertoxin I (ATX-I). AOH, AME, and TEN belong to the chemical group dibenzo-α-pyrones, representing the major *Alternaria* toxins, whereas TeA, ALT, and altertoxins such as ATX-I are considered emerging *Alternaria* mycotoxins and belong to the amine metabolite, benzopyrone, and perylenequinone chemical classes, respectively [4]. These compounds have demonstrated several toxicological properties, including cytotoxicity, genotoxicity, mutagenicity, and immunomodulatory effects in in vitro and in vivo models [5,6,7,8,9], raising concerns regarding their safety and potential impact on public health. Nevertheless, regulatory frameworks addressing *Alternaria* mycotoxins remain underdeveloped, largely due to a scarcity of data on their occurrence and toxicokinetics in biological systems.

Due to its role in the metabolism and detoxification of xenobiotics, the liver is considered a key target organ for mycotoxin toxicity [10]. The potential bioaccumulation of *Alternaria* mycotoxins in hepatic tissues poses significant concerns for both human and animal health, as it may result in chronic toxicity, including hepatic injury, carcinogenesis, or disruption of metabolic functions [7,11].

However, data concerning the presence and accumulation of *Alternaria* toxins in liver tissue remain largely unexplored. Most of the existing research has focused on the detection of mycotoxins in biological fluids such as plasma and urine [12,13] and other matrices such as capillary blood or feces [13], which do not provide direct information on tissue distribution and organ-specific toxicodynamics.

The liver tissue is characterized by its biochemical complexity, which requires the use of robust and efficient sample preparation techniques to be employed to successfully isolate and concentrate mycotoxins. Among the sample treatment procedures applied for the determination of mycotoxins in biological tissues, solid–liquid extraction (SLE) has been applied, followed by liquid–liquid extraction (LLE) or salt-assisted LLE (SALLE) [14,15,16], as well as the ‘QuEChERS’ procedure for edible animal tissues such as chicken muscle and liver [17,18]. More recently, dispersive liquid–liquid microextraction (DLLME) preceded by SALLE or SLE has been successfully used to determine multiple mycotoxins in both human and animal liver and other biological tissues [19,20]. Hence, salt-assisted extraction techniques represent effective strategies to enhance the determination of mycotoxins in biological tissues.

To date, no studies have reported the determination of *Alternaria* mycotoxins in liver samples or other animal tissues, representing a critical gap in food safety research. In this context, the present study aims to develop and validate a comprehensive analytical methodology based on SALLE followed by liquid chromatography coupled with quadrupole time of flight (Q-TOF) mass spectrometry for the simultaneous determination of six key *Alternaria* mycotoxins, AOH, AME, TeA, TEN, ALT, and ATX-I in human and animal liver samples. The untargeted LC-HRMS acquisition of the main *Alternaria* toxins would allow the exploration of other metabolites derived from these mycotoxins, providing a comprehensive overview of their metabolism.

## 2. Results and Discussion

### 2.1. Optimization of Sample Treatment

The most effective extraction technique and the best conditions for the isolation of the *Alternaria* mycotoxins of interest were investigated at this step. The optimization of sample treatment was achieved through the employment of suckling lamb liver procured from a local supermarket in Murcia, Spain. Samples from other species were analyzed only to evaluate the applicability of the method. Prior to the optimization of sample treatment, the material was examined to ensure its mycotoxin-free status. Blank status was evaluated by analyzing the material using the method of Castell et al. (2023) [19], confirming the absence of mycotoxins. Then, it was spiked with AOH, AME, TEN, TeA, ALT, and ATX-I standards at a concentration of 100 µg kg^−1^.

**Figure 1:**
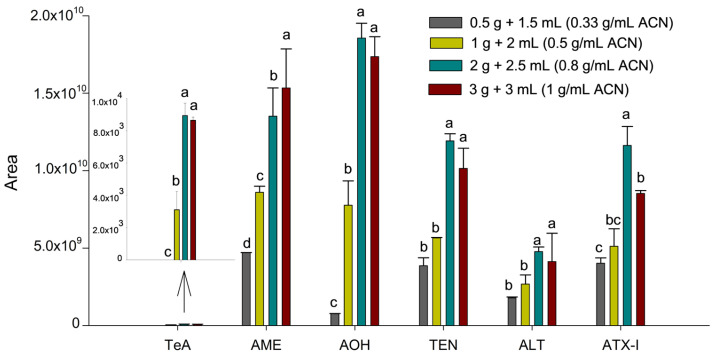
Influence of the sample-to-solvent ratio on the sensitivity of the *Alternaria* mycotoxins. Results are expressed as mean integrated area (*n* = 2, independent extractions) ± standard deviation. Different letters (a, b, c, d) above the bars for the same analyte indicate statistically significant differences (one-way ANOVA followed by Tukey’s post-hoc test, *p* < 0.05).

Initially, the SALLE protocol described by Castell et al. [19] was applied to the tissue sample, as this salt-assisted extraction technique has proven effective for mycotoxin determination in complex matrices. The optimization of the extraction procedure was then performed by evaluating parameters such as sample amount, solvent volume and type, and salt concentration. First, to optimize the influence of sample amount and solvent volume (sample-to-solvent ratio), homogenized suckling lamb liver samples of 0.5, 1.0, 2.0, and 3.0 g were spiked at 100 µg kg^−1^ with the six *Alternaria* mycotoxins. For all conditions, 3.0 mL of water where added, whereas the volume of ACN was set to 1.5, 2.0, 2.5, and 3.0 mL, respectively, resulting in sample-to-solvent ratios of 0.33, 0.5, 0.8, and 1.0 g mL^−1^, considering ACN volume. Thus, the amount of analyte was proportional to the sample mass, and peak areas were compared to evaluate the extraction efficiency trend at a fixed concentration (100 µg kg^−1^). This allowed us to identify the point where increasing the sample-to-solvent ratio no longer produced a proportional increase in signal, indicating potential solvent saturation or matrix interference. ACN was used as the reference solvent as it is the most commonly used solvent in SALLE procedures and provides an appropriate baseline for optimizing extraction ratios. Each condition was evaluated using two independent extractions (injected twice)**,** and the results are expressed as mean integrated area ± standard deviation (Figure 1).

As can be seen in Figure 1, an increase in the ratio from 0.33 to 0.8 g mL^−1^ of ACN resulted in a substantial enhancement in peak areas for all toxins (*p* < 0.05). However, a subsequent increase to 1.0 g mL^−1^ of ACN (3.0 g of liver) led to a decline or no change in the response for specific analytes like ATX-I (*p* = 0.0028). This decline in efficiency at the highest ratio suggests a saturation of the extraction solvent and a concomitant increase in co-extracted matrix components from the liver. Therefore, the optimal threshold of 0.8 g mL^−1^ (2.0 g of liver and 2.5 mL of ACN) was determined to achieve the greatest sensitivity, while ensuring extraction yields were satisfactory and matrix interference was minimized.

Then, the nature of the organic solvent employed was optimized to enhance both phase separation and the extraction efficiency of mycotoxins, especially those exhibiting low recovery rates, maintaining the previously optimized sample-to-solvent ratio. For this purpose, samples were prepared using different solvents: MeOH, acetone, ACN, and EA. It was found that MeOH and acetone failed to produce a clear separation between the organic and aqueous phases, not enabling the extraction of mycotoxins or giving rise to high variability, respectively. In contrast, ACN and EA demonstrated effective phase separation, attributed to their lower miscibility with water, thereby facilitating the recovery of the organic layer.

Comparisons between ACN and EA revealed better extraction efficiency for TeA, AME, and AOH when using EA, whereas no differences were observed for the remaining mycotoxins. Accordingly, EA was selected as the extraction solvent. Prior to the optimization of ionic strength on extraction efficiency, the selected solvent volume was re-evaluated using EA to confirm that the sample-to-solvent ratio remained appropriate after changing the extraction solvent. It was confirmed that the use of 2.5 mL of EA ensured efficient phase separation and consistent extraction performance, as no significant differences were observed among the tested values, thus supporting the suitability of this solvent volume.

**Figure 2:**
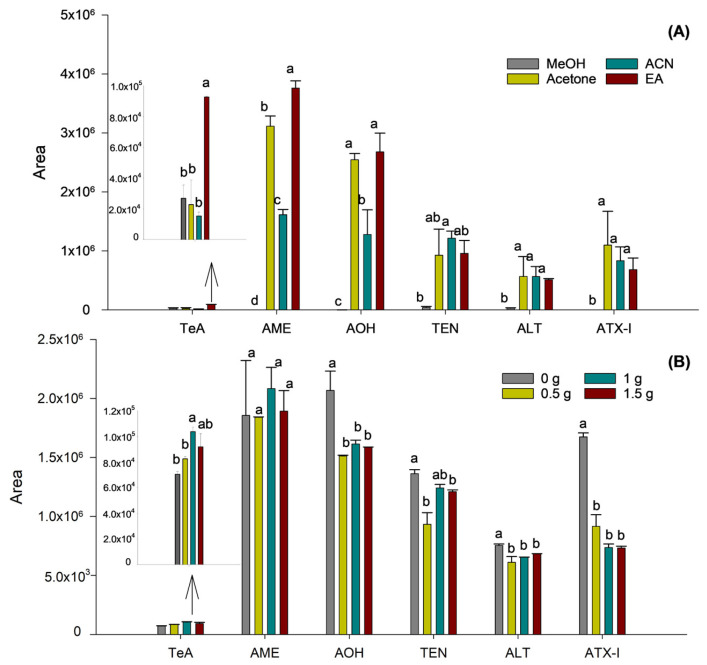
Influence of the extraction solvent (**A**) and addition of NaCl (**B**) on the sensitivity of the *Alternaria* mycotoxins. Results are expressed as mean integrated area (n = 2, independent extractions) ± standard deviation. Different letters (a, b, c, d) above the bars for the same analyte indicate statistically significant differences (one-way ANOVA followed by Tukey’s post-hoc test, *p* < 0.05).

As a final step in optimizing the SALLE procedure, the effect of varying the amount of NaCl on extraction efficiency was evaluated. To this end, four duplicate samples were prepared with NaCl quantities ranging from 0 to 1.5 g, in 0.5 g increments. As shown in Figure 2B, although the condition without salt showed a tendency towards higher responses for AOH and ATX-I, the addition of 1 g of NaCl significantly improved the extraction of the TeA mycotoxin (*p* = 0.0245).

In contrast, for the remaining mycotoxins, no significant differences in extraction efficiency were observed across the tested salt concentrations. Thus, as TeA appeared to benefit particularly from the effect of ionic strength using salt, the addition of 1 g of NaCl was selected as the optimum.

### 2.2. Validation of the Method

A summary of all validation results is presented in Table 1.

Initially, the matrix effect was evaluated by means of signal suppression/enhancement (SSE) magnitude. For this purpose, a comparison between slopes of calibration curves after performing linear calibration in a lamb liver blank matrix or in a neat solvent was carried out. Therefore, the SSE effect was calculated as SSE (%) = 100 × (slope of calibration curve in spiked matrix extracts/slope of calibration curve in neat solvent). As shown in Table 1, SSE varied between 53% for TeA and 78% for AME, evidencing a signal suppression in the presence of matrix (values below 100%). Because of the matrix effect observed for certain investigated mycotoxins, the employment of matrix-matched calibration was considered essential for precise quantification.

Calibration curves were constructed by spiking suckling lamb liver samples at seven concentration levels, and regression coefficients (R^2^) exceeded 0.99 for all mycotoxins, indicating excellent linearity within the examined concentration ranges. For the proposed method, the LOD values ranged from 0.01 (AME) to 1.46 (TeA) µg kg^−1^, and LOQs were between 0.04 and 4.85 µg kg^−1^ for AME and TeA, respectively.

Trueness, repeatability, and intermediate precision were assessed at three concentration levels. Level 1 (lower level) and Level 2 (intermediate level) corresponded to 0.1 and 1 µg kg^−1^ for AME, AOH, and TEN, while for TeA, ALT and ALT-I were set at 6 and 10 µg kg^−1^, respectively. Level 3 (high level) was established at 20 µg kg^−1^ for AME, AOH, and TEN, while for TeA, ALT, and ALT-I it was set at 60 µg kg^−1^. Suckling liver samples (2.0 g) were fortified with the corresponding level of concentration and left for 30 min equilibration prior to extraction following the optimized procedure.

Trueness, expressed as apparent recovery, was calculated as the ratio of measured concentration to the actual (spiked) concentration multiplied by 100. Each concentration level was prepared in triplicate. Recoveries ranged from 84% to 111%, with relative standard deviation (RSD) values for each mycotoxin provided in parentheses (see Table 1). These results comply with Commission Regulation 2023/2782 [21], which establishes laboratory control standards for mycotoxin analysis.

Precision was evaluated through repeatability and intermediate precision. Repeatability was determined by analyzing triplicate (instrumental replicates) samples spiked at the three concentration levels within the same day (experimental replicates), while intermediate precision was assessed by repeating the experiments across three different days. The obtained RSD values were below 10%, confirming the appropriate precision of the method.

Supplementary Figure S1 showed the extracted ion chromatograms obtained for a fortified suckling liver sample (20 µg kg^−1^ for AME, AOH, and TEN and 60 µg kg^−1^ for TeA, ALT, and ATX-I) using the developed and validated procedure using UHPLC-HRMS. Detailed information regarding precursor *m*/*z* values and retention times is provided in the Supplementary Table S1.

### 2.3. Analysis of Liver Samples

The presence of *Alternaria*-derived mycotoxins in hepatic tissue was determined by analyzing twenty human liver samples from twenty different autopsies and twenty animal liver samples (5 pig liver, 5 calf liver, 5 chicken liver, and 5 rabbit liver) by the described method. Samples were prepared in duplicate and treated by the SALLE procedure described for the extraction and preconcentration, then subsequently injected into the UHPLC-QTOF-MS system. QTOF mass spectrometry selection over a triple quadrupole instrument was motivated by the dual objective of the study: quantitative determination of selected *Alternaria* mycotoxins and exploratory screening of additional *Alternaria*-derived metabolites.

As can be seen in Table 2, AME was detected in only one pig liver sample at a concentration of 3.1 ± 0.3 µg kg^−1^ (Table 2). In contrast, the mycotoxins AOH and TEN were exclusively found in human forensic liver samples. TEN was the most frequently detected Alternaria mycotoxin, present in two samples with similar concentrations ranging from 1.86 ± 0.04 to 2.22 ± 0.05 µg kg^−1^, while AOH was quantified in one sample at 2.5 ± 0.2 µg kg^−1^.

The analysis of liver samples from other species was carried out to demonstrate the applicability of the proposed methodology. Overall, the detected mycotoxins exhibited comparable concentration levels, with AME showing slightly higher values than AOH and TEN. None of the other *Alternaria* mycotoxins included in the study (ALT, TeA, and ATX-I) were detected in any of the analyzed liver tissues.

The liver samples analyzed in this study were not selected based on documented dietary or environmental exposure to *Alternaria* mycotoxins. Consequently, the detected concentrations should be interpreted as exploratory findings rather than as indicators of population-level exposure or food safety risk. At present, no regulatory limits have been established for *Alternaria* mycotoxins in biological tissues, which precludes a direct risk-based interpretation of the measured levels.

Nevertheless, the liver tissue is a key organ for xenobiotic metabolism and accumulation, and several *Alternaria* mycotoxins, including AOH, AME, and TEN, have been reported to exhibit hepatotoxic, genotoxic, and immunomodulatory effects in vitro and in vivo [5,6,7,8,9]. Although the oral bioavailability of most *Alternaria* toxins is generally considered low, experimental studies indicate that certain compounds may persist in hepatic tissue or undergo metabolic activation. Therefore, the detection of these mycotoxins in liver samples, even at low concentration levels, supports the relevance of investigating their tissue distribution.

Overall, these results demonstrated the applicability of the proposed analytical methodology to real liver matrices. Future studies specifically designed to evaluate exposure scenarios, toxicokinetics, and bioaccumulation patterns will be required to establish the toxicological significance of *Alternaria* mycotoxins in liver tissue and their potential implications for food safety.

### 2.4. Alternaria Mycotoxin Metabolite Analysis. Untargeted Approach

At last, to achieve a more comprehensive evaluation of *Alternaria* toxin metabolite occurrence in liver samples, additional research was conducted on the presence of previously described derivatives and conjugates for which reference standards were not available. Specifically, apart from the six main *Alternaria* mycotoxins studied, 13 metabolites derived from *Alternaria* genus, belonging to different chemical classes and derived from *Alternaria* genus, were examined using an untargeted approach. These included dibenzopyrones, perylene quinones, nitrogen containing compounds, other phenolic compounds, as well as several phase-II metabolites previously described [9,22,23], all listed in Table 3.

The data acquired through the DDA method employing auto MS/MS mode within a 100–1000 *m*/*z* range was further processed, beginning with the extraction of molecular entities using MassHunter Profinder 10.0 software. Molecular feature validation followed strict criteria described in Section 4.6 for isotopic distribution evaluation. This approach guarantees the identification of features corresponding to the same potential metabolite by considering specifically the information of the deprotonated ion ([M-H]^−^) along with the neutral loss by dehydration and the acetic acid adduct ([M+CH_3_COO]^−^). Consequently, molecular entities obtained, characterized by using their retention time, accurate mass measurement, and intensity of the chromatographic peak, were identified by applying the custom database outlined in Table 3.

After all, apart from the three previously monitored and validated mycotoxins, no *Alternaria*-derived metabolites were found in liver samples, whether human or animal.

Although a direct comparison between ESI-positive and -negative ionization modes under salt-free conditions could provide additional insights into ionization efficiency and additional information for the untargeted approach, the scope of the present work focused on employing a single and robust workflow for *Alternaria* toxins that is applicable to both targeted and non-targeted analyses. This approach was selected to ensure method consistency and reproducibility. However, it is acknowledged that the addition of salt during sample preparation can result in the formation of sodiated adducts. This has not been considered, as only the ESI-negative mode was employed. Nevertheless, future studies should include a systematic evaluation of ionization efficiency under salt-free conditions to further expand the applicability of the methodology.

## 3. Conclusions

This study presents the first validated method for simultaneously determining six *Alternaria* mycotoxins in human and animal liver tissues based on SALLE coupled to UHPLC-QTOF-MS. Achieving an efficient and reproducible sample preparation protocol was made possible by optimizing SALLE conditions, including the evaluation of sample mass, extraction solvent, and ionic strength. The method was validated in spiked suckling lamb liver samples, fulfilling the requirements established by current regulatory guidelines in terms of linearity, trueness, and precision. The applicability of the method was demonstrated through the analysis of real liver samples of both animal and human origin, with AME being detected in pig liver and AOH and TEN in human liver tissues. Although no other target *Alternaria* mycotoxins or additional metabolites were detected, these findings highlight the potential bioavailability and hepatic presence of selected *Alternaria* toxins. The reported concentrations should be interpreted as exploratory, as the method was fully validated in a representative liver matrix using spiked samples and not under controlled exposure conditions. Nevertheless, due to its straightforward sample preparation and adequate analytical performance, the proposed methodology represents a useful analytical tool for investigating the occurrence of *Alternaria* mycotoxins in liver tissue. Furthermore, this approach may contribute to generating the occurrence data needed to support future toxicokinetic studies and to improve understanding of the tissue distribution of Alternaria mycotoxins in humans and animals.

## 4. Materials and Methods

### 4.1. Reagents and Standards

All reagents used in this work were analytical grade, and solvents were chromatographic grade. Acetonitrile (ACN), ethyl acetate (EA), acetone, and methanol (MeOH) were obtained from Chem-Lab (Zedelgem, Belgium), while chloroform (CHCl_3_) was provided by Sigma-Aldrich (St. Louis, MO, USA), as was the sodium chloride (NaCl) used for sample treatment.

The water used was ultrapure quality with a resistivity greater than 18.2 mΩ/cm obtained using a Milli-Q system (Millipore, Bedford, MA, USA). Syringe filters (0.2 μm × 13 mm) from Agilent Technologies were used to filter the samples.

The individual Alternaria mycotoxin standards (AOH, AME, TEN, TeA, ALT, ATX-I) were purchased from Romer Labs Diagnostic GmbH (Tulln, Austria). Standard solutions at 100 mg L^−1^ in ACN were prepared and stored at −20 °C.

### 4.2. Instrumentation and Software

The UHPLC system employed for sample analysis was composed of an Agilent 1290 Infinity II Series HPLC accompanied by a high-speed binary pump (Agilent Technologies, Santa Clara, CA, USA) and a ZORBAX RRHD Eclipse Plus C18 column (2.1 mm inner diameter, 1.8 μm particle size, and 100 mm length) in conjunction with a 0.3 μm in-line filter from Agilent Technologies. The detection process was conducted using an Agilent 6550 Q-TOF mass spectrometer (Agilent Technologies, Santa Clara, CA, USA), which was equipped with an Agilent jet stream dual electrospray ionisation (AJS-Dual ESI) source (Agilent Technologies, Santa Clara, CA, USA).

The extracted ion chromatograms (EICs) thus obtained were then recorded and processed using Profinder 10.0 and MassHunter Qualitative Analysis B.07.00 softwares (Agilent Technologies, Santa Clara, CA, USA). Further data processing was conducted by Excel software (Microsoft Office 365 ProPlus, Washington, DC, USA).

The samples were homogenized and stored individually at −20 °C until analysis. During the sample processing stage, an EBA 20 centrifuge from Hettich (Tuttlingen, Germany), an LLG-uniTEXER vortex mixer from Heathrow Scientific (Vernon Hills, IL, USA) and an XcelVap air drying system from Horizon Technology (Salem, MA, USA) were used.

SigmaPlot 16.0 (Grafiti GmbH, Düsseldorf, Germany) was used for data representation and statistical analysis.

### 4.3. Liver Samples

A total of twenty liver samples from pigs, lambs, chickens, and rabbits were procured from local markets in Murcia, Spain. Another twenty human liver specimens were collected from eight autopsies conducted at the Institute of Legal Medicine in Murcia (Murcia, Spain). All samples were homogenized and stored separately at −20 °C until further analysis.

For the human liver samples, the following data were available: age, sex, cause of death, and manner of death. The cohort consisted of eighteen males and two females, aged between 29 and 86 years. Deaths were classified as suicides, natural causes, or accidents, with specific causes including asphyxiation, gastrointestinal haemorrhage, cardiovascular disease, and cranial trauma. The study was conducted in compliance with data protection regulations and applicable legislation, and in accordance with the ethical standards approved by the Ethics Committee of the University of Murcia (protocol number 1848/2018).

### 4.4. Sample Treatment

A modified analytical procedure consisted of applying the SALLE technique described by Castell et al. [19], which yielded an organic phase containing the analytes. Liver homogenization was performed prior to weighing by using a blade homogenizer. Approximately 10 g of tissue from each liver was minced and homogenized for 2 min until a uniform paste was obtained. The homogenized material was then transferred to sterile containers and used as the source material for all analytical subsampling. Aliquots of 2.0 g were subsequently weighed from this homogenized batch for extraction and placed into a 15 mL plastic centrifuge tube. Three millilitres of ultrapure water followed by 2.5 mL of EA were added to the sample, and the mixture was vortexed for 10 s after the water addition and for 2 min after the organic solvent addition. Next, 1.0 g of NaCl was added, and the mixture was shaken vigorously for 1 min and then centrifuged for 5 min at 4500 rpm, allowing the organic phase containing the mycotoxins (approximately 2 mL of EA) to separate. The organic supernatant collected was filtered through a double 0.2 μm nylon filter and stored in a glass vial for subsequent injection into the UHPLC-QTOF-MS system. All samples were prepared in duplicate.

### 4.5. UHPLC-QTOF-MS Analysis and Method Validation

Chromatographic separations were performed using a UHPLC-QTOF-MS system. The elution of the analytes was carried out using a gradient program at a flow rate of 0.4 mL/min, with the following mobile phases: solvent A (H_2_O:ACN, 95:5, *v*/*v*) and solvent B (ACN:H_2_O, 95:5, *v*/*v*). Both solvents contained 0.5% acetic acid (*v*/*v*).

The elution gradient consisted of a linear increase of solvent B from 0% to 60% over 12 min, followed by a ramp to 100% in 1 min, a return to initial conditions of 0% in 1 min, and a final 2 min isocratic hold at 0% solvent B. The total separation time was 16 min, and 20 µL were used as the injection volume. Column temperature was set at 30 °C.

The electrospray ionization (ESI) source operated in negative mode with the following instrument parameters: 130 °C gas temperature, 16 L/min gas flow, and 30 psi nebulizer pressure, 300 °C sheath gas temp, 11 L/min sheath gas flow. The scan source operated voltages of 4000 V, 360 V, and 750 V corresponding to capillary, fragmentor, and octupole. The auto MS/MS mode was used to perform a data-dependent analysis (DDA) for MS detection in the range *m*/*z* 100–1000. Acquisition mode MS Scan rate was 5 spectra/sec with medium isolation width MS/MS (~4 amu) and 20 V as fixed collision energies. Data were acquired in centroid mode. The *m*/*z* range was autocorrected on reference masses 112.9855 and 1033.9881. Identification criteria of *Alternaria* mycotoxins consisted of achieving: mass accuracy ≤ 5 ppm, isotopic pattern match ≥ 80%, retention time within ±0.15 min, and at least two characteristic MS/MS fragments of the one observed in standards.

The validation of the proposed method was carried out by evaluating its linearity, limits of detection (LOD) and quantification (LOQ), trueness, precision (intermediate precision and repeatability), and matrix effect (signal suppression enhancement). Calibration curves were constructed by spiking suckling lamb liver samples at seven concentration levels (between 5 and 100 µg kg^−1^ for TeA, ALT, and ATX-I and between 0.1 and 60 µg kg^−1^ for AOH, AME, and TEN, preparing three replicates of the spiked concentration levels. Linearity was assessed by calculating the regression coefficients (R^2^) and LODs, and LOQs were determined using the signal-to-noise ratio (*S/N*) criteria of 3 and 10, respectively. Trueness, repeatability, and intermediate precision were assessed at three concentration levels (low, intermediate, and high) and were expressed as relative standard deviation (RSD).

### 4.6. Data Processing and Statistical Analysis

MassHunter Qualitative Analysis B.07.00 was employed for the processing of the data acquired by UHPLC-QTOF-MS in auto MS/MS mode. The process of treating the raw data file commenced with the extraction of potential molecular features (MFs) using Profinder, which was facilitated by the suited algorithm incorporated in the software. In the context of the recursive extraction algorithm, all ions exceeding 5000 were categorized as the cut-off frequency. Furthermore, the isotopic distribution to consider a molecular feature as valid should be defined by two or more ions with a peak spacing tolerance of *m*/*z* 0.0025 and a mass accuracy of 5.0 ppm. Besides the deprotonated [M−H]^−^ ions, the formation of adducts in the negative ionization mode, together with selected neutral losses, was taken into account to group signals corresponding to the same metabolite. These related signals were subsequently treated as single entities characterized by their retention time, accurate mass, and peak intensity. After signal alignment, chromatographic peaks were integrated to generate a curated data matrix, which was exported in CSV format for the identification of MFs using an online or custom database. A detailed summary of the EIC integration and filtering parameters applied in Profinder is provided in Supplementary Table S2.

The integrated chromatographic data were then subjected to statistical evaluation using Sigmaplot 16.0 software. Analysis of variance testing (one way-ANOVA) was applied to assess statistical significance (*p* ≤ 0.05), followed by Tukey’s HSD post hoc test to determine significant differences in the relative concentration of identified compounds.

## Figures and Tables

**Table 1:** Method validation data for *Alternaria* mycotoxin determination in liver samples.

Mycotoxin	Linear Range (µg kg^−1^)			Linearity, R^2^	LOD ^a^ (µg kg^−1^)	LOQ ^a^ (µg kg^−1^)
AME	0.04–60			0.989	0.01	0.04
AOH	0.06–60			0.988	0.02	0.06
TEN	0.05–60			0.996	0.02	0.05
TeA	4.85–100			0.992	1.46	4.85
ALT	3.35–100			0.993	0.99	3.35
ATX-I	3.85–100			0.992	1.16	3.85
	**Trueness, % RSD**					**SSE (%)**
	**Level 1**			**Level 2**	**Level 3**	
AME	88.2 (4.7)			91.5 (5.2)	89.7 (1.4)	78
AOH	94.4 (7.9)			84.4 (8.3)	86.1 (5.6)	60
TEN	90.5 (2.3)			86.8 (2.6)	92.7 (2.1)	75
TeA	109.6 (3.8)			110.8 (1.9)	97.9 (2.5)	53
ALT	92.1 (6.6)			93.4 (3.2)	103.8 (1.7)	80
ATX-I	97.4 (2.9)			99.4 (2.8)	108.6 (6.5)	65
	**Repeatability, % RSD**			**Intermediate precision, % RSD**		
	**Level 1**	**Level 2**	**Level 3**	**Level 1**	**Level 2**	**Level 3**
AME	6.5	7.0	5.7	7.9	8.1	6.5
AOH	8.2	9.9	2.8	8.7	9.4	6.2
TEN	2.7	2.3	3.9	4.7	5.2	5.8
TeA	6.8	6.9	4.0	7.9	5.9	5.9
ALT	7.8	9.2	1.8	9.9	9.7	7.6
ATX-I	4.9	9.6	4.6	6.6	8.7	5.4

LOD, limit of detection (*S*/*N* = 3), LOQ, limit of quantification (*S*/*N* = 10); SSE, magnitude of signal suppression/enhancement. Level 1: 0.1 µg kg^−1^ AME, AOH and TEN; 6 µg kg^−1^ TeA, ALT and ATX-I. Level 2: 1 µg kg^−1^ AME, AOH and TEN; 10 µg kg^−1^ TeA, ALT and ATX-I. Level 3: 20 µg kg^−1^ AME, AOH and TEN; 60 µg kg^−1^ TeA, ALT and ATX-I. ^a^ LOD and LOQ were estimated using the following quantifier ions: 196.0977 (TeA), 271.0612 (AME), 257.0461 (AOH), 291.0886 (ALT), 351.0879 (ATX-I), 413.2177 (TEN).

**Table 2:** *Alternaria* mycotoxins detected in liver samples with positive findings.

Samples	Concentration (µg kg^−1^)					
	TeA	AME	AOH	TEN	ALT	ATX-I
Pig liver	ND	3.1 ± 0.3	ND	ND	ND	ND
Human liver 1	ND	ND	2.5 ± 0.2	2.22 ± 0.05	ND	ND
Human liver 2	ND	ND	ND	1.86 ± 0.04	ND	ND

ND: not detected.

**Table 3:** *Alternaria* metabolites researched.

No	[M-H]^−^ Mass	Compound Name	Molecular Formula	Abbreviation
Dibenzopyrones				
1	257.0455	Alternariol	C_14_H_10_O_5_	AOH
2	271.0611	Alternariol Monomethyl Ether	C_15_H_12_O_5_	AME
3	291.0874	Altenuene	C_15_H_16_O_6_	ALT
4	291.0874	Isoaltenuene	C_15_H_16_O_6_	isoALT
Perylene quinones				
5	351.0874	Altertoxin I	C_20_H_16_O_6_	ATX-I
6	349.0718	Alterperylenol	C_20_H_14_O_6_	ALP
7	349.0718	Altertoxin II	C_20_H_14_O_6_	ATX-II
8	347.0561	Stemphyltoxin III	C_20_H_12_O_6_	STTX-III
9	347.0561	Altertoxin III	C_20_H_12_O_6_	ATX-III
N-containing compounds				
10	413.2194	Tentoxin	C_22_H_30_N_4_O_4_	TEN
11	196.0979	Tenuazonic Acid	C_10_H_15_NO_3_	TeA
12	398.2337	Altersetin	C_24_H_33_NO_4_	ATS
Phenolic compounds				
13	289.0718	Altenusin	C_15_H_14_O_6_	ALS
14	323.0772	Altenuic acid III	C_15_H_16_O_8_	AA-III
Phase-II-metabolites				
15	433.0776	AOH-3-*O*-glucuronide	C_20_H_18_O_11_	AOH-3-Gluc
16	447.0933	AME-3-*O*-glucuronide	C_21_H_20_O_11_	AME-3-Gluc
17	433.0776	AOH-9-*O*-glucuronide	C_20_H_18_O_11_	AOH-9-Gluc
18	337.0024	AOH-3-*O*-sulfate	C_14_H_10_SO_8_	AOH-3-S
19	351.0180	AME-3-*O*-sulfate	C_15_H_12_SO_8_	AME-3-S

## Data Availability

The original contributions presented in this study are included in the article/Supplementary Materials. Further inquiries can be directed to the corresponding author.

## Supplementary Materials

The following supporting information can be downloaded at: https://www.mdpi.com/article/10.3390/toxins18020077/s1, Table S1. UHPLC-HRMS parameters of the target *Alternaria* mycotoxins; Table S2. Peak integration and filtering parameters used for UHPLC-QTOF-MS data processing in MassHunter Profinder; Figure S1. Extracted ion chromatograms obtained for a fortified stuckling liver sample (20 µg kg^−1^ for AME, AOH and TEN and 60 µg kg^−1^ for TeA, ALT and ATX-I) using the developed and validated SALLE coupled to UHPLC-HRMS procedure; Figure S2. MS spectrum results of targeted *Alternaria* mycotoxins.

## Abbreviations

The following abbreviations are used in this manuscript:

AA-III	Altenuic acid III
ACN	Acetonitrile
AGREEprep	Analytical Greenness Metric for Sample Preparation
AJS-Dual ESI	Agilent jet stream dual electrospray ionization
ALS	Altenusin
ALP	Alterperylenol
AME	Alternariol monomethyl ether
AME-3-Glc	AME-3-glucoside
AME-3-S	AME-3-sulfate
AOH	Alternariol
AOH-3-Glc	AOH-3-glucoside
AOH-3-S	AOH-3-sulfate
AOH-9-Glc	AOH-9-glucoside
ATX-I	Altertoxin I
ATX-II	Altertoxin II
ATX-III	Altertoxin III
ATS	Altersetin
BAGI	Blue Applicability Grade Index
CHCl_3_	Chloroform
DDA	Data-dependent analysis
DLLME	Dispersive liquid–liquid microextraction
EA	Ethyl acetate
ESI	Electrospray ionization
LLE	Liquid–liquid extraction
LOD	Limit of detection
LOQ	Limit of quantification
MeOH	Methanol
MS	Mass spectrometry
NaCl	Sodium chloride
ND	Not detected
Q-TOF	Quadrupole time of flight
RSD	Relative standard deviation
SALLE	Salt-assisted liquid–liquid extraction
SLE	Solid–liquid extraction
SPMS	Sample Preparation Metric of Sustainability
STTX-III	Stemphyltoxin III
TeA	Tenuazonic acid
TEN	Tentoxin
UHPLC	Ultrahigh-performance liquid chromatography
