# Supplementary material for: Development and Validation of an LC-MS/MS Method for the Determination of Alternaria Mycotoxins in Hepatic Tissue
Source: Toxins (Basel). 2026 Feb 2;18(2):77. doi: 10.3390/toxins18020077 (PMC12945079; doi:10.3390/toxins18020077)

# Development and Validation of an LC-MS/MS Method for the Determination of *Alternaria* Mycotoxins in Hepatic Tissue

**Table S1.** UHPLC-HRMS parameters of the target *Alternaria* mycotoxins.

| Compound | RT[a] (min) | Precursor ion (*m/z*) | Product ion 1 (*m/z*) | Product ion 2 (*m/z*) |
|---|---|---|---|---|
| TeA | 7.57 | 196.0977 | 139.0273 | 128.9945 |
| AME | 11.29 | 271.0612 | 256.0380 | 204.9900 |
| AOH | 8.80 | 257.0461 | 213.0556 | 189.0557 |
| ALT | 7.31 | 291.0886 | 186.9991 | 166.9927 |
| ATX-I | 8.50 | 351.0879 | 315.0671 | 333.0765 |
| TEN | 8.90 | 413.2177 | 271.1447 | 214.0733 |

[a] RT: retention time

**Table S2.** Peak integration and filtering parameters used for UHPLC-QTOF-MS data processing in MassHunter Profinder.

| Parameter | Setting / Description |
|---|---|
| Feature extraction mode | Batch Targeted Feature Extraction |
| Peak integration type | Extracted Ion Chromatogram (EIC)-based |
| Signal used for integration | Parent precursor ion ($MS^1$) |
| Integrator selection | Agile 2 |
| Smoothing function | Gaussian |
| Isotopic pattern integration | All isotopes grouped per molecular feature |
| Match tolerance masses | ±5 ppm |
| Match tolerance retention time | ±0.15 min |
| Minimum peak height | 5000 counts |
| Chromatogram data format | Centroid when available, otherwise Profile |
| Peak width | Automatically determined by Profinder |

**Figure S1.** Extracted ion chromatograms obtained for a fortified stuckling liver sample (20 μg $kg^{-1}$ for AME, AOH and TEN and 60 μg $kg^{-1}$ for TeA, ALT and ATX-I) using the developed and validated SALLE coupled to UHPLC-HRMS procedure.

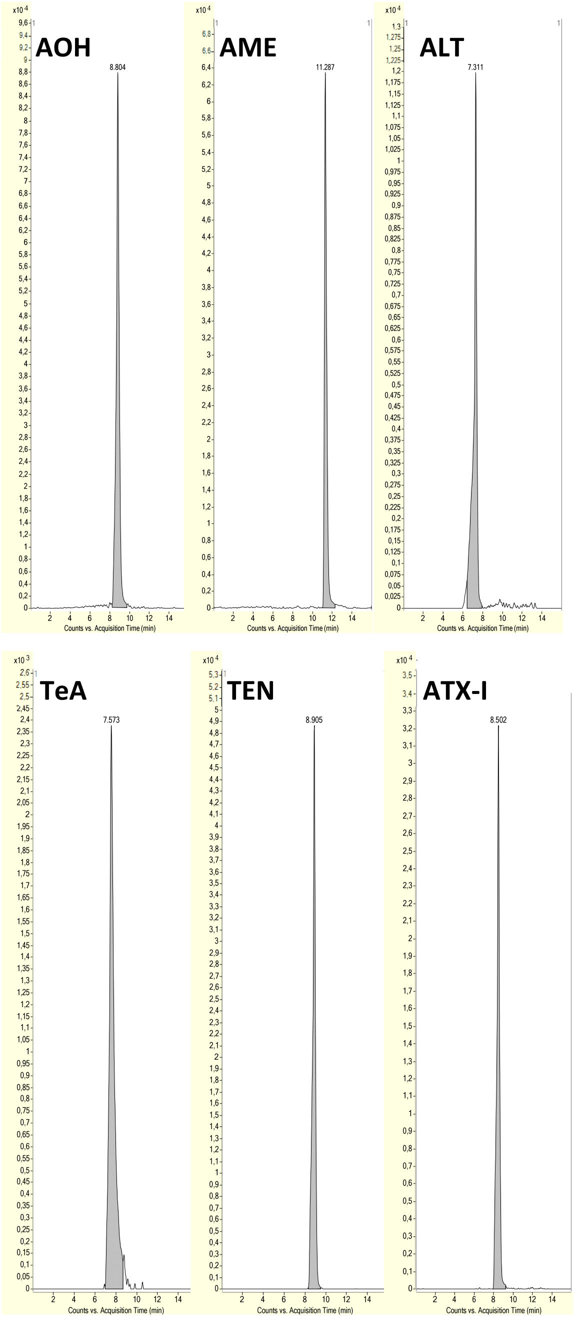

**Figure S2.** MS spectrum results of targeted *Alternaria* mycotoxins.

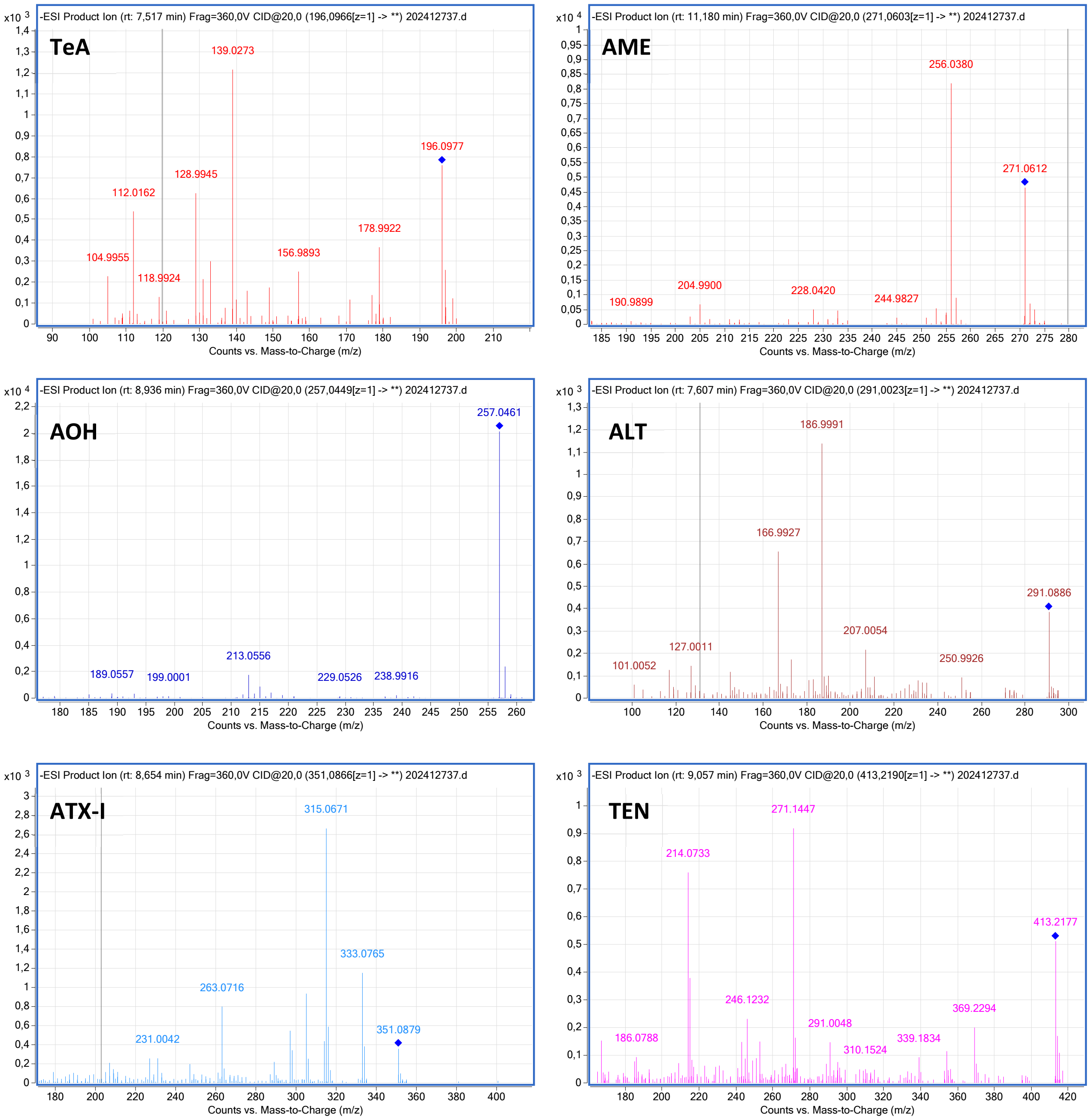

Supplement: Supplementary file 1 [file toxins-18-00077-s001.zip › toxins-4021438-supplementary.pdf]
